# The Hospital Nursing Supplement

**Published:** 1893-02-18

**Authors:** 


"The Hospital" Nursing Mirror.

Being the Extra Nursing Supplement of "The Hospital" Newspaper.

[Contributions for this Supplement should be addressed to the Editor, The Hospital, 140, Strand, London, W.C., and should have the word "Nursing" plainly written in left-hand top corner of the envelope.]

### News from the Nursing World.

### THE EMPRESS FREDERICK.

On the day preceding her departure from Berlin to visit the Queen at Osborne, the Empress Frederick paid a visit to Freiderichshain. In 1884 seven trained nurses from the "Victoriahaus" first went to work there, taking charge of certain pavilions, and in 1892 the numbers had increased to fifty-five nurses and probationers. The Empress Frederick takes a constant and practical interest in both nurses and their work, and pays frequent visits to the institution named after her, as well as to the large city hospital, Friederichshain.

### DELIGHTFUL ASSURANCE.

Many experienced women are volunteering opinions on the question of young nurses, but a quotation from a letter written by a girl applicant gives yet another view of the subject: "Although I have not had any previous training in hospital work, I have a perfect knowledge of all that is required of me, and am quite sure I could perform my duties satisfactorily."

### WORKERS WANTED.

Many young ladies have not much money at their command, but a large number have several hours in each day which they could give away. Time is as precious an offering as money, and yet the fact is but little acknowledged. Single women whose home duties do not claim more than a small part of the day, can well devote a proportion of the surplus to helping over-worked women and frail children. District visiting is not always desirable, and certainly is not the duty of every-body, but useful work should occupy part of all women's lives. Such labours of love are their own reward, for custom makes them easy and "the demand" eventually secures "the supply." At the Children's Hospital, 2, Maida Vale, the demand for helpers far exceeds the supply at present, but perhaps that is only because that modest little home is not as well known or understood as it ought to be. The inmates are chiefly incurable, and many of the poor little people are helpless. They need amusing and feeding and nursing. Not in the ordinary way common to childhood, but to a much greater extent. Inexperienced people would think that each of these little sufferers would need a separate nurse, but training proves otherwise. The staff of regular workers is a small one, and there is no room to accommodate more, even if the funds permitted of extra nurses. The matron, who "mothers" these poor little people, has an admirable plan of allowing voluntary day helpers to come and assist in taking care of her "family." Not only are the ladies welcomed as play-fellows and instructresses, but they are also initiated into regular nursing details. A qualified staff nurse overlooks their work, and many young ladies would gladly avail themselves of this opportunity of gaining useful experience, if they paid but one visit or else wrote to Miss Coleman, 2, Maida Vale, for details of her plan. A gracious welcome is given to all earnest workers, and these are sorely needed at present. Ladies who only want to pass an idle hour by "looking on" simply hinder busy people. Steady helpers are the "workers wanted" just now.

### COMFORTS IN SICKNESS.

The Lancaster Ladies' Nursing Society are discussing the possibility of indulging in a second district nurse. They have only one at present, and this is not sufficient for the needs of a town containing over 31,000 inhabitants. There are now twenty cases on the books, all under the care of Nurse Elizabeth Wilson, whose services are highly valued by her patients. Miss Wilson was trained at Preston Infirmary, and received a certificate there. The Committee of the L.L.N.S. also want subscriptions towards a "Sick Comfort Fund" for the supply of beef-tea, eggs, &c. In such a large town as Lancaster modest wants such as these should be promptly supplied.

### NURSING AT TUNBRIDGE WELLS.

A public meeting was lately held at Tunbridge Wells in connection with the District Nursing Association, and the doctors all spoke most warmly of the value of trained nurses. An interesting speech was made by Miss Louisa Twining, who described the success which had attended the movement at Kensington. Miss Warner, who is a well-known district nurse at Tunbridge Wells, gave the results of her experience amongst the poor in a graphic manner. Her terse summary of efficient nursing was excellent—"My idea of good nursing centres in this, the maximum of comfort given with the minimum expenditure of the patient's strength."

### MIND AND BODY.

It is an acknowledged necessity that nurses should receive a complete course of training in each branch of their profession. Instruction in every kind of general nursing can be obtained at many excellent schools attached to hospitals and infirmaries. Some special diseases, however, are still unknown, save in name, to probationers at the end of a three years' course. Many kinds of brain affections are never seen in ordinary wards; or, at any rate, they are not long detained there, for obvious reasons. Nurses who are constitutionally and intellectually fitted for taking up the important work commonly known as "mental," need, in addition to a full general training, a special course of instruction on the treatment of such patients. Good nurses are always in demand for cases, which, however they may vary in degree, invariably require the most skilful and tender care. Reason as well as life may largely depend on the intelligence with which a nurse carries out the doctor's orders and treatment. Granted the need for instruction, where can it be obtained? Dr. Greene, of Berry Wood Asylum, Northampton, has resolved to give probationers a chance of sharing in the advantages of training, for which the institution under his charge gives such unusual facilities. To receive, train, and give certificates to satisfactory candidates is the new programme which he has laid down, and the accommodation and general comforts of the nurses have been adequately provided for. The scheme is one which deserves, and doubtless will command eventual success. Dr. Harding's course of lectures and practical instruction are already arranged, and nurses anxious to obtain the benefit of this exceptional opportunity for special training at moderate fees should lose no time in applying to the Medical Superintendent, Berry Wood, Northampton.

### FAULTY SANITATION.

A capital meeting was held lately by the Society for Providing Nurses for the Sick Poor of Belfast. The ladies connected with it appear as practical as they are enthusiastic, and certainly the report of the General Superintendent is a valuable one. She says: 'Our efforts to instil sanitary precautions into the minds of those amongst whom we work are unceasing. . . . We cannot help feeling that improvement in the heavy death-rate cannot be expected until we have a daily thorough cleansing of all the streets and lanes, and a more effective supervision of back premises. Dirt appears to us the factor to blame for much of the disease from which we suffer in Belfast. . . . Our working classes are well fed and well housed, and with a little care the reproach of an abnormally high death-rate might be removed." Such candour should certainly appeal strongly to medical officers and sanitary authorities at Belfast, and it should induce them to alter the existing evil by banishing the uncleanness which is the origin of "preventable disease," and of many wasted human lives.

### PROGRESS AT LONDONDERRY.

Londonderry District Nursing Society, which is affiliated with the Q.V.J.I., has increased and prospered amazingly. The nurses are credited with the improvements which have taken place in the homes they visit. "Cleanliness and sanitary reforms have been introduced by them, thus making the houses of the poor more healthy, and helping to stamp out diseases that otherwise might reach our own homes," says the annual report. The Bishop of Derry made an admirable speech at the meeting, stating that the nurses acted from beginning to end on medical advice, so far as it was given. He amused his large audience by referring to the liking for medicine not ordered by the doctor, which he had observed amongst the people in the North of Ireland. He spoke very fully of the nurses' duties, and the way in which they fulfilled them, and asked for increased subscriptions to provide, amongst other things, an additional nurse to undertake night duty, of which his lordship truly estimates the importance. Applause followed the words, "As sickness is unsectarian, this ministry of healing should be unsectarian too."

### NURSING AT SYDNEY.

The new Nurses' Home at Sydney is connected with the Prince Alfred Hospital by a covered way, and it stands in a pleasant part of the grounds. It is altogether worthy of the beautiful hospital of which it forms an adjunct, being a handsome building and perfect in every department. A room is specially set apart for the nurses' practical lessons in cooking, besides fine sitting and work rooms, sick room, library, &c., the latter well furnished with books. There are thirty-five single rooms and five double ones for the accommodation of the nursing staff, and all are exceedingly well furnished. The plans for the Home were submitted to Miss Nightingale, who has shown her deep interest in the nurses by presenting a bust of herself to the institution. The Home was formally opened by the Countess of Jersey, who was presented by Miss McGahey, the Matron, with a handsome basket of flowers. The Prince Alfred Hospital authorities are to be congratulated on having secured the services of Miss Susan McGahey, a lady whose administrative capacity and training especially fit her to discharge successfully the important duties of a Superintendent of Nursing.

### SHORT ITEMS.

The courses of lectures by members of the medical staff to the probationers at the Mater Misercordiæ Hospital, Dublin, have been resumed. Fully-trained certificated nurses wishing to join the Nurses' Co-operation at the next election can obtain forms of application from the Lady Superintendent on applying personally between ten a.m. and one p.m. at the office, 8, New Cavendish Street.—The Guardians have granted an additional nurse to the workhouse infirmary at York, although fourteen gentlemen voted against the innovation, thirty-four were fain to confess that a hundred patients to each of the present nurses was too heavy a responsibility. The existing arrangement also necessitates these over-worked day nurses being called up at night to attend to the lying-in ward.—There was rather a sensational meeting recently of the St. Austell Board of Guardians, and in the course of the proceedings the workhouse nurse claimed the protection of the Local Government Board "from the abuse and insults of the master." Her letter had been submitted to the clerk before being sent to the Local Government Board, "but the Guardians took no action in the matter." The Visiting Committee tendered their resignation, and mentioned various matters that had come under their notice, but felt "the impossibility of doing any good under existing circumstances." Although persuaded to reconsider their decision, they seem justly dissatisfied with present arrangements.

### A DISABLED NURSE.

The following donations have been received during the week, in company with many kind expressions of sympathy for the disabled nurse: Nurse Bruce collected 3s. 6d.; Cambridge nurses, 5s. 6d.; Policy 79, 2s. 6d.; J. R. D., 2s.; S. A. Y., 10s.

### "THE HOSPITAL" ENDOWED BED.

Subscriptions for the nurses Bed are now due. All contributions sent to The Editor, The Lodge, Porchester Square, will appear in The Hospital. We have pleasure in acknowledging £1 from A. B.; Policy 79, 2s. 6d.; J. R. D, 2s.

### Management of Consumption.

### II.—SYMPTOMS AND SIGNS OF CONSUMPTION.

Consumption is most commonly met with during the earlier periods of life. To be precise, more cases are met with between the ages of 15 and 35 than at any other time. Its ravages are most manifest where people are crowded together in unhealthy dwellings, with insufficient food for daily requirements. Where the population is scattered one does not so often meet with the disease. In some towns the mortality from this complaint is terrible. In Glasgow, for instance, during some years it has come up to half the total deaths, and in Vienna, Brunn, and Prague, still more appalling figures have been obtained.

For purposes of description we may distinguish three kinds of consumption, acute, sub-acute, and chronic. Before describing these, we will, however, give a general account of the signs of the disease. For the most part consumption begins gradually and insidiously. The patient does not notice much the matter, except that he feels more tired after his work, and perhaps for some time he has had a slight hacking cough without any expectoration. Very soon, though, definite symptoms declare themselves. The cough, which before had scarcely been noticed, becomes worse, and may be attended with slight frothy expectoration, the patient finds that his appetite, which before was robust, is not so good now, and he has an aversion to fatty articles of diet. He finds at the same time his clothes do not fit him as before, and on weighing himself generally learns he has lost a considerable amount of flesh. He also notices at night that his sleeping gown is damp with perspiration. He frequently has a pain more or less persistent of a stabbing and lancinating character at one or the other apex. When he exerts himself at work his breath gets very short, and he soon becomes unfit for hard work. In the evening his cheeks are flushed, especially over the cheek bones. The expectoration has now become more copious, and is thick and of a nummular character. Sometimes, without any warning while at work, or sitting still, he has a profuse hæmoptysis, bringing up, perhaps, two pints of blood or more. With the accession of these grave symptons all history of a former cough and expectoration is forgotten by the patient, and he frequently adheres to the statement that until the blood-spitting came on he was in the enjoyment of good health. Sooner or later he is forced by failing health to consult a medical man, who elicits the foregoing symptons from him more or less complete. If the disease be established on looking at the patient's chest, the following points will be noticed: The chest will be emaciated, the rib spaces easily seen, under one collar bone the chest will be flattened more than on the other side. On taking a deep breath it will be seen that one side of the chest moves much less than the other; there is impairment of movement. If the side of the chest which is flattened be percussed with the fingers it will yield a duller sound than the other. The points determined by use of the stethoscope do not much concern us here, suffice it to say that by it still further evidence of consumption having attacked the lungs is obtainable, and it can be made out how far advanced the disease is, whether a cavity has already formed, and how far the disease extends in the lungs.

This, then, is the general picture consumption presents at its beginning; under treatment the disease may improve considerably, and in some cases be completely arrested, but in the majority of people, especially among poor people, a time comes sooner or later, sometimes in a few months, sometimes in two years, when they break down again. The old symptoms return with increased severity and less prospect of improvement, until at last they become advanced consumptives, with both lungs hopelessly disorganised and only a few weeks of life remaining.

In the acute form of consumption all the symptoms come on with great rapidity; there is no gradual onset; there is sweating, emaciation, dyspnœa of a severe character, cough, expectoration, high temperature, and profound weakness from the first; often there is diarrhœa; both lungs become rapidly diseased, the illness never takes a turn for the better, and death takes place in from six weeks to three or four months.

The sub-acute variety is very similar, but is generally not so rapid, and though progressing steadily to a fatal termination, remissions are not infrequent.

In the purely chronic variety the patient may go on for years without the disease making any marked progress. Often in winter time he is brought up short with an extension of the disease, but under appropriate treatment this rapidly subsides and he again enjoys a fair measure of health.

Everyone with cough and expectoration has not consumption, but all consumptives suffer from cough and expectoration. People with dyspepsia have a cough, and bring up a slight amount of phlegm; they also lose flesh to a certain extent, and frequently have a pain in the left side. It is these symptoms which fill the out-patient departments of chest hospitals to overflowing with dyspepsia, indeed with people who would fain believe themselves consumptive.

A few words may be said about the treatment of the disease. There is no known specific for consumption. All drugs from time to time brought forward as a cure have failed to bear the test of a thorough trial. Our means for arresting consumption lie in the care with which we attend to the general health of the patient, and the assiduity with which we look after his powers of digestion. We must, at all costs, improve his nutrition and build him up, so that he may resist his disease. To this end we give him fresh air, freedom from worry and over exertion. We improve his general health by means of tonics, such as strychnine, phosphoric acid, iron, arsenic, and quinine. We look after his digestive functions by giving him the different stomactics such as gentian, bicarbonate of soda, nitro-hydrochloric acid, calumba, and many others. We at the same time give him food which, while being readily assimilable, will also tend to fatten him; chief among these comes cod liver oil, and years of patient investigation have failed to bring to light anything superior to this best of foods. Cream, milk, and plain wholesome food in general form the diet on which the consumptive is most likely to thrive. By these means we not infrequently see the disease arrested and the patient rescued from what looked like certain death. On the other hand, cases come under treatment too late for this method to be of any service, and our best efforts are met with failure.

*(To be continued.)*

### Minor Appointments.

Miss Blanche Smith has been appointed assistant to the lady superintendent at the Norfolk and Norwich Hospital.

Miss E. Cann has been appointed Night Sister at the Hospital for Women, Soho Square. Miss Cann was trained at the South Devon and East Cornwall Hospital, Plymouth, and afterwards held the post of Sister of the Gynæcological ward there.

Miss Margaret G. Halkett has been appointed Assistant Matron at Mill Road Infirmary, Liverpool. Miss Halkett was trained at the London Hospital, and afterwards filled the post of matron's assistant. Her departure is sincerely regretted by her old friends, and we cordially join in their good wishes for her success in her new work.

Miss Key has been appointed District Nurse by the Woolton District Nursing Society. She was trained at the Brownlow Hill Infirmary, Liverpool, and afterwards worked for two years in the infirmary of St. George's in the East. Miss Key also holds the diploma of the L O.S. We wish her every success in her new sphere of usefulness.

### Our Examinations.

THE question set for February has proved a most popular one, nearly a hundred answers having been received. Not only is the number satisfactory, but the kind of replies sent in denote much thoughtful intelligence. Very few indeed can be entirely condemned, and a great many merit praise. Most writers are thoroughly imbued with the need for isolation, but we must warn them against the somewhat arbitrary measures which too many seem, at any rate on paper, inclined to practise. It is very well to say, "I should at once place the patient in a room at the top of the house," but we are disappointed to find not a single nurse has apparently taken into consideration the obvious fact that the patient to whom they are summoned may be sleeping in a room which, although not on the top, which is undoubtedly the best floor for the purpose, may yet be conveniently placed for nursing purposes, and, moreover, the apartment being already infected is an important consideration in houses of moderate capacity. Many papers not only decree this immediate removal, but they want two additional rooms set apart, which, with the chamber in which the patient first fell ill, would considerably reduce the accommodation of an ordinary dwelling. Mansions, of course, can spare a whole suite of rooms, but we want our nurses to show us how well they can arrange for the complete isolation of one patient without giving avoidable discomfort to the other members of the household.

Nearly everyone has given intelligent consideration to food, mentioning the need for burning everything left by the patient. Only one or two answers, however, suggest the wisdom of only taking into the sick-room the quantity likely to be consumed at a time. One nurse speaks of giving the pieces of food left, wrapped up in paper, "to the servant to burn," but we hope she does not practise such rashness. However, other writers suggest sending infected things down to the kitchen, which is certainly the last place in the house where they ought to be deposited. We are glad to observe that the majority of nurses grasp their personal responsibilities in cases of infectious disease, and most of them, though not all, give intelligent descriptions of the daily cleaning of the room with disinfectants, not with dry dusters. We wish more competitors had given the strength of the disinfectants they recommend, instead of leaving it to the imagination. Washing dresses for the nurse are insisted on in almost every case, but only one or two persons are thoughtful enough to say "and washing petticoats," and the recommendation that skirts should be short is in most cases omitted. The common-sense of the last hint is so obvious that we can only wish it were universally followed.

With regard to inunction and baths, a grave error occurs in more than half the papers, but we trust it is an accidental one, and not in any way a type of the writers' own procedure, for the number and frequency of the baths is dictated, without any reference to the doctor. A more serious error in a disease so subject to "complications" cannot be imagined, and the medical man's responsibility in the matter can never be too clearly realized. The inunctions should be, of course, equally dependent on his wishes. With regard to personal disinfection, the answers are on the whole very good and thorough. "Stoving" the rooms afterwards is well described by only a few nurses, the majority treating the subject as vaguely as they do the strength of the disinfectants. Ventilation, again, is insisted on to almost an alarming extent. We must warn our friends to avoid that dangerous substitute for intelligent ventilation, which consists in creating a thorough draught; the room should be aired without any undue airing of the patient. With regard to the disinfection of bed and body linen, only two writers mention the important precaution of bringing the pail with the disinfectant to the linen instead of carrying the latter to the receptacle. We conclude with a point which nearly all our competitors have commenced with, namely, the sheet over the door, which is to prove such a safeguard to the rest of the household. Many lay emphasis on the necessity for keeping it wet, and suggest methods of making it so; only one gives any hint to facilitate permanent dampness. Now we should like careful consideration to be given to this by no means unimportant detail. When the case is in the hands of one nurse it is essential that some method should be practised which would obviate any necessity for her having constantly to drench the sheet which gets dry with such amazing rapidity. We invite remarks on this subject, and shall be pleased to insert the best letter we receive.

The prize has been awarded to Nurse Weedon, whose answer we give:—

"What precautions against infection should be taken during and after the nursing of a case of scarlet fever in a private house?"

The patient should be isolated; a room at the top of the house is the best; bed hangings, curtains, and carpet should be removed, also any needless furniture and useless articles. A sheet should be hung over the door outside and kept moist with carbolic (1 in 20). No sweeping should be done, the floors and furniture should be wiped over once every day with a cloth wrung out of carbolic lotion. Crockery, &c., must be kept specially for the patient's use, and nothing should be taken out of the room without first being disinfected. All the remains of food should be burnt. The discharges from throat and nose should be wiped away with rag and burnt at once. The patient should be rubbed from time to time with carbolized oil or ointment (subject to doctor's orders). A basin containing carbolic should be kept in the room, into which the nurse should dip her hands every time after touching patient. The nurse should keep strictly to her own apartments, and before going to any other part of the house or out of doors she should wash her face and hands with carbolic soap and change her dress; it is better to change all the clothes, and, if possible, to take a bath. As soon as the period of desquamation is over, patient should have a bath (including head) in water in which some disinfectant has been added, and should put on clothes that are free from infection. After the room is vacated, all bed and body linen and washable articles should be thrown into a pan containing carbolic (one pint of crude acid to two gallons of water is a very good solution for the purpose). After soaking for several hours, they should be boiled. All flannels, blankets, and bedding which cannot be boiled should be thoroughly opened and spread out. All cupboards and drawers should be opened, the chimney should be blocked, and all cracks and crevices pasted up. One pound of sulphur should be placed in an iron pot (1½ lb. of sulphur is needed for a large room); a little spirits of wine should be added. It should be placed in the centre of the room, supported over a pail of water for safety. The spirits of wine should then be lighted (a live coal may be used instead of the spirits of wine if preferred), and the room door securely closed and any cracks pasted up. The room should be left for at least eight hours, and then the window should be opened and the chimney cleared to allow free ventilation. Before the room is used again, the floor and furniture should be scrubbed with carbolic (one pint of crude acid to three gallons of water) and the wall papers stripped off and the walls and ceiling limewashed. The nurse should thoroughly disinfect herself before leaving.

### Appointments.

MISS MABEL ANDERSON, late of St. Mary's Hospital, Paddington, has been appointed Lady Superintendent of the Meath Home for Epileptics, Godalming. Miss Anderson first took the post on trial for six months, and the committee passed a cordial vote of thanks for her excellent organization, and economical management.

MISS AMY JOHNSTONE has been appointed Matron of the Infirmary at Salisbury. Miss Johnstone was trained at the Royal Infirmary, Liverpool; was afterwards night superintendent at St. Marylebone Infirmary; held a similar post at Charing Cross Hospital, and then became Matron of Boston Hospital, Lincolnshire. Her testimonials are excellent, and she takes with her the good wishes of a great many friends.

### Croupous Pneumonia.

Paper read before the Nurses' Journal Club at the Training School for Nurses of the Johns Hopkins Hospital, April, 1892.

*(Continued from page cxlvi.)*

BUT as to the pneumonia itself—how is infection received, and what goes on after it has taken place?

The air is undoubtedly an important source of infection in pulmonary diseases, though now in the treatment of wounds no longer greatly dreaded. The pneumococcus may exist in floating dust in the air, but this has not been often observed, and it is an open question whether or not pneumonia is often acquired by inhaling the specific germ of the disease. The germ may be found habitually present in the mouth in states of greater or less virulence, in a number of persons ranging from twenty to fifty per cent. It is then easy to imagine its progress down the respiratory tract into the lungs. But things are made as hard for these germs as possible. It is not an easy matter to enter the lung substance. Even when this is accomplished the bronchial glands stand ready to take up bacteria which are not capable of growing in living tissue; but the air cells themselves present the chief opposition.

Dr. Welch's description of the structure and characteristics of the cell walls, as designed by nature, and as found in a state of perfect health, was so beautiful and brilliant that I wish it were not impossible to reproduce it here. However, the impression left on the mind after hearing it, was that these marvellous tissues were intended to be, and should be, proof against any invasion.

They combine the most exquisite and gossamer-like delicacy with the most astonishing resistive power. Inert foreign bodies, such as coal-dust, can be safely stored away in enormous quantities in their recesses, so securely that the regular work of breathing is but little or not at all interfered with thereby. Specimens of "coal miner's lung" were shown in this connexion, blackened with the deposit of years, yet uninjured.

To many forms of bacteria they are absolutely impermeable. Perhaps to all they would be, but for the "accessory causes," without which no bacterial disease can take root.

The accessory causes are all those which were once believed to be the true ones; exhaustion, alcoholism, exposure to cold and wet and others. All act alike, whatever they are. Exorbitant in their demands on the vitality of the tissues, the latter pay out more than they get in. Their resistive power is weakened, their structural integrity is impaired and they are open to attack. The bacteria—omnipresent—find their opportunity; they enter, and begin growth. This is the end of the first act.

To begin the second some new characters must be introduced.

First, the pneumotoxine, the poison produced by the organisms in their growth.

Next, the white blood corpuscles or leucocytes which play so important a part, and the chemotactic substance which calls out the leucocytes from their homes.

Finally, the anti-pneumotoxine, the antidote provided in the blood to meet the poison, and whose saving efforts culminate at the time of the crisis.

The micro-organism of pneumonia, like some others that we know of, is not terrible in itself, but in the poison that it produces while growing. It is this poison, this pneumotoxine, which, entering the blood, is accountable for the prostration that we see in the patient, and for the frequent death by heart failure.

If it be allowed to circulate unchecked through the system the patient is speedily overwhelmed and t e heart stopped by paralysis of its nerve supply.

What will happen now? Shall the struggle be ended at the very outset by an easy victory for the bacteria? By no means. The body tissues declare war, and the first step taken is the erection of a barricade to block up the invaders.

The soldier-like qualities of the white corpuscles need not be explained now, as all have heard or read something of their part in inflammatory processes. Ordinarily present in the blood in small numbers, as compared with the red, their native haunts are in bone marrow and out-of-the-way places; but when called upon for action they flock out in immense numbers, climb through the cell walls, and quickly reach their destination. In the present instance this is, of course, the lungs, and a gradually solidifying exudation fills up the lung at the point threatened; a mass that under the microscope is seen to consist of fibrin, detritus from cell walls, red corpuscles, and white ones, and effectually walls in the micro-organisms and cuts off their further advance into the circulation. We thus see the consolidation of the lung in pneumonia to be, not a pernicious, but a protective condition; not a malign process, to be attacked and doctored off if possible, but a defence against bacterial poison; a stronghold representing the resistance of the tissues to an enemy.

Otherwise it would stand thus: The more consolidation, the graver symptoms; but as a matter of fact the severity of the symptoms bears no constant relation to the amount of consolidation. On the contrary an imperfect leucocytosis leaves open the path into the circulation, and there is more blood poisoning, more prostration and correspondingly bad prognosis.

*(To be continued.)*

### "Victoriahaus fur Krankenpflege."

THE institution which bears this title has grown into a most important one. At the present time it occupies an independent and definite standpoint in Berlin, and provides for the nursing of 17 hospitals and clinics.

The training of probationers is done chiefly in the large hospital known as "Friederichshain," and is on the general lines followed at St. Thomas's School, the Lady Superintendent being herself a "Nightingale. Probationers must be between the ages of 25 and 35, must hold medical certificates of good health, and be of good education. Their applications must be accompanied not only by letters of recommendation, but by an account of their own previous history written and composed by themselves. The nominal training is for only one year, but each candidate on entering signs an agreement promising to remain for two years after her training is completed, and to serve as private, district, or hospital nurse.

Two courses of lectures are given by doctors during the year, and the Lady Superintendent is responsible for the practical instruction of the probationers. She lives in the house with the latter, and takes dinner and supper in their company in the large dining-hall. The other meals are served to the probationers in the sitting-rooms adjacent to the wards, and all the food provided is said to be excellent.

Each ward sister has a separate bed-room and a sitting-room, and most of the nurses have separate bed-rooms. The probationers are lodged in large airy dormitories curtained off into cubicles.

The day sisters and nurses are on duty fourteen hours daily, and the probationers thirteen hours, one hour for recreation being granted in addition to the meal times, and once in the week each member of the nursing staff has a half day off duty.

Each nurse has about four months' night duty during the year, taking only four weeks at a time. After the year of probation has expired, every nurse is entitled to a month's holiday annually.

The Empress Frederick is not only a constant visitor, but it is greatly due to her zeal and energy that the nursing staff has attained dimensions which enable it now to provide skilled attendants for all classes of the community.

### Everybody's Opinion.

*[Correspondence on all subjects is invited, but we cannot in any way be responsible for the opinions expressed by our correspondents. No communications can be entertained if the name and address of the correspondent is not given, or unless one side of the paper only be written on.]*

### USEFUL HINTS WANTED.

"AN INTERESTED NURSE" writes: I think it would be helpful if some amongst the thousands of readers of THE HOSPITAL would find time to write down some of their experiences in other countries. For instance, many Englishwomen have been in America, and it would help those who want to go this year if some nurse with experience of living and travelling in the country would tell us about it. It is hard for persons unaccustomed to travelling to guess at what things cost elsewhere. A little sensible information would be welcomed by those who, like myself, feel uncommonly ignorant of other countries.

### THE BEST NURSES.

NURSE FRANCIS says: Miss Hampden's letter is most interesting, and we are all very glad to have had the chance of reading it. The Americans seem inclined to make a big thing of the Nursing Department, and certainly they are taking a wide view of the subject and inviting papers on a splendidly inclusive scale. I do hope our best English nurses, best in every way, will rise to the occasion. The worst of it is, good nurses are generally so busy that they think their individual duties exclude all public ones. But this being a special time and a particular occasion, surely arrangements might make it comparatively easy for a few really representative nurses to be sent over to Chicago. We should all be contented to leave our interests in the hands of those whom experience has shown to be our best friends. Perhaps the Editor will kindly encourage suggestions on this subject.

### ENGLAND *VERSUS* AMERICA.

"A. E. I." writes: After reading the reports of the lecture given by Mrs. Bedford Fenwick, on January 20th, I cannot refrain from making a few observations. She says that nursing is a profession in America, not a trade, as in England. Certainly, Americans are fond of high-sounding words, but if Mrs. Fenwick means to imply that they work in America with higher aims than in England, she is most certainly mistaken. The almighty dollar is quite as powerful in the nursing profession of America as any love of gain is in England, and many nurses I have spoken to in America frankly owned that they had no love for the work, but had to make their living, and nursing was "a well-paid profession." Now, amongst English nurses I have generally found love for the work existing, even if they are obliged to gain a livelihood by it. It is also well-known that many Englishwomen have been successful heads of hospitals in America. Miss Alice Fisher, at Philadelphia, is an instance, and she and her friend did much for the nursing world there. Her death is still deeply lamented. All American doctors and employers allow that English nurses are reliable, and hard workers. In the course of three years' private nursing in America I was frequently told that it was not usual for ladies to be nurses. Class distinctions in America are hard to define, and all education being free, even the daughters of the poor are educated, in a certain sense of the word. Mrs. Fenwick said that probationers in England receive salaries, and yet the governing body reserves the right of discharging them, and she calls this "a one-sided arrangement." Does she mean to imply that the probationers should also have the right to discharge the governing bodies? In New York the probationers are sent out from the hospitals, private nursing, during their first year, at a fee of 16 dollars a week, they themselves receiving about 14 dollars a month—16 dollars is four less than fully-trained nurses receive. The registry offices for trained nurses charge enormous fees. As regards food, three meals a day for patients and nurses, the last about six or seven p.m., in America, do not seem to me to compare favourably with English hospital diets. In the hospital in America, where I worked for some months, neither doctors nor nurses had ever seen a properly-marked four-hour chart, although some of the latter had come from other institutions. They warmly approved the ones I drew out, and had copies printed for future use.

### THE LATE MR. HEWER.

"A PRIVATE NURSE" writes: May I ask you, on behalf of other nurses and myself, to find space in THE HOSPITAL for a few words with regard to the late Mr. Hewer, of Highbury. We all feel that in him we have lost a kind and true friend, and one we cannot easily replace. He never came into a sick room without leaving not only the patient, but also the attendants, cheered and comforted by his visit. He never thought of himself, but would come early and late to see his patients, and, as you truly said, "his patients never left him." Two years ago I nursed a case for him in Essex, and though he had done a hard day's work in town and was not well himself at the time, every evening at eight o'clock he would regularly appear, and, thanks to his skill, the patient recovered. From personal experience, I can bear witness to Mr. Hewer's unceasing kindness to nurses. He did not regard us as mere necessaries in a sick room, but took a real interest in us, and in times of sickness, either from overwork or other causes, or if we were in trouble, we could always rely on his kind help and sympathy. Mr. Hewer is a universal loss; a doctor like him cannot be replaced. He has truly laid down his life for his patients, and all who ever came in contact with him mourn their loss, and feel deep sympathy for all his family. May I correct a slight mistake with regard to Mr. Hewer's age? He passed away on the day before his 62nd birthday.

### WORKHOUSE INFIRMARIES.

NURSE EMILY writes: If you have space in THE HOSPITAL, I should like to answer "Nurse W.'s" questions on infirmary nursing. I have been for three years a nurse at St. Saviour's Infirmary, East Dulwich, and can answer her questions from personal experience. (1) Off Duty Time (day nurses)—Three hours every other day and every third Sunday; one day's holiday a month, and three weeks annually. Twenty-four hours on changing from day to night duty. (2) The convalescent inmates do not assist in the nursing, but do light work, such as sweeping the wards, dusting, &c., which helps to lighten the nurse's work considerably. (3) Extra help is always given to assist with bedridden cases, operations, double pneumonia, &c. (4) The assistant nurses are moved at the Matron's discretion. Food is good, and we are all thoroughly well nursed and attended to when ill.

### "THE HOSPITAL" ENDOWED BED.

NURSE J. R. D. writes: I send 2s. for the endowed bed. If every reader of THE HOSPITAL would only give 1s., what a help it would be! What is one bed? We need one for the North, as well as for the South, the East, and the West. I also enclose 2s. for the disabled nurse, with sincere sympathy, and hopes for her complete restoration to health. Would that she had been in the R.N.P. Fund for sick pay, as I was. It is only those who have benefited from being on the fund who can feel duly grateful to the promoters of it.

### The History of Nursing.

Notes of an introductory lecture given by Dr. SHAW to the nurses of the Bristol Royal Infirmary.

(*Continued from page* cxlvii.)

FROM about A.D. 400 to A.D. 1000, that is, during the first half of the Middle Ages, nursing was practised in Europe in monasteries and nunneries alone, viz., Augustine and Benedictine houses. The nursing orders were called "Infirmarii" or "Hospitalarii." They treated as well as nursed the sick, there being scarcely any doctors in Europe at the time. The Abbess Hildegarde of Bingen wrote a work on popular remedies.

From about 1000 A.D. to 1500 A.D, or the second half of the Middle Ages, was a period of pilgrimages, crusades, and charitable institutions. As the large towns developed special hospitals were founded—already in the sixth and seventh centuries the two Hotels Dieu at Paris and Lyons had been founded.

A.D. 1123.—St. Bartholomew's Hospital in London was founded.

On the pilgrims' routes special inns ("hospices") were established in many places, where were nursed any pilgrims who might be sick; at some hospices there were "Sisters" (Dames de qualité) for the nursing of the pilgrims. The still existing Hospice of St. Bernard in the Alps was founded A.D. 980.

A.D. 1206.—Pope Innocent III. founded the hospital "San Spirito," at Rome, after which, in Milan and other towns, hospitals were founded of great magnificence, some by nursing orders, others by charitable individuals, and others by town corporations. The nursing was provided by the nursing orders. Subsequent to A.D. 1100, men, women, boys, and girls placed themselves under the guidance of the Church, wore a religious dress, took the vows of celibacy and obedience, and dedicated themselves to perpetual nursing.

From A.D. 1209 the Franciscans have dispersed as a nursing order over nearly all the countries of the world.

These "hospitalarii" were some burgher and some noble, and, from presents made to them, the greater number of the orders became very rich between the twelfth and fifteen centuries. After the fifteenth century many of the *male* nursing orders were abolished on account of misconduct and misuse of their riches. The *female* nursing orders have remained faithful.

The most important nursing order of the Middle Ages was that of St. John.

About A.D. 1100 a pilgrims' inn was established at Jerusalem by the Order to entertain and nurse the pilgrims. Gradually this institution increased greatly in extent and wealth, so that the members of the order were called the Knights of St. John of the Infirmary at Jerusalem. In the twelfth century this hospital was the largest in the world; 2,000 poor and sick were daily fed and nursed there. The Order instituted hospices in England and most other European countries, and continued to be a nursing order until they were removed from Rhodes to Malta in 1522.

Many other nursing orders besides those already mentioned were instituted in the Continent of Europe previous to the year 1500, but were not connected with nursing in this country. All mediæval hospitals, it must be remembered, were refuges for the poor, aged, orphans, widows, &c., as well as for those really sick, and, therefore, corresponded to the modern workhouse, as well as the modern hospital. The only special hospitals which existed were the leper houses, which existed in great numbers during the Middle Ages. In the twelfth century it is estimated that there were 19,000 of them in Western Europe. Doctors were only exceptionally found in the hospitals of the Middle Ages.

(*To be continued*).

### For Reading to the Sick.

### THE STAR.

WE read in the Bible that when God created the stars He said, "Let them be for signs." For centuries they were used by storm-tossed mariners to guide them to the haven where they would be. As children we were deeply interested by the "sweet story of old," which told how three wise men steered their course by a star through the darkness of heathen ignorance to the light of Christ made manifest in the flesh. These astronomers, whose business it was to scan the heavens and make their calculations of the changes of seasons, when summer and winter, seed time and harvest would take place, some nineteen hundred years ago saw a star of singular brilliancy which they had never observed before, and as they gazed and gazed the meaning of its presence was borne in on their minds; they knew that it was sent to lead them to the new-born King, the Saviour of the World. So these three men took counsel together and followed where they were led. They felt called to leave the happiness of home, to go out into an unknown world, to meet the scorn and derision of friends, to be called dreamers and fanatics. Their neighbours would warn them that they would perish by the way, but they heeded not, their faces were set to find that better country, though, alas! when found, many disappointments awaited them. To their inquiries, "Where is the King?" they could get no answer; nobody knew aught about Him, and when at last Herod, for his own evil purposes, sent them into the presence of Christ, the stable for His home, the manger for His cradle, must have been great trials to their faith, but the Magi worshipped and adored.

We must not lose in the interest of the story the beautiful lessons it teaches. Many of us have been guided to Christ by the signs which he has given us; lights in the darkness they have been, though at first we did not recognise them. Sorrows, sicknesses, crosses, these shine out brightly in the lives of most Christians, as the stars which have led them to Christ. Like the Magi, we have passed through much tribulation before we could rejoice with exceeding great joy. "Your sorrows shall be turned into joy" is our blessed Lord's promise to those who seek and find Him. If our sorrows have been the instruments by which God has led us to Himself, we shall understand the meaning of Christ's words, "Blessed are they that mourn, for they shall be comforted."

The friendships of life are often true stars to guide us to Christ. How greatly the influence of a good man or woman has led us heavenward; what comfort and sympathy and encouragement is there for a feeble soul, when those that fear the Lord speak often one to another and never fail to pass on the message, "The Master is come and calleth for thee." It is the loneliness of the way which appals to us, though we have a "friend which sticketh closer that a brother," yet He allows us to find comfort in the star of human love, where, as in St. John's case, it is pillowed on the Divine Breast.

We read that by God's direction the Magi returned home another way. Probably by an easier road, for their faith having been tried and established, it only remained for them by their example to strengthen the brethren.

### Wants and Workers.

The Parish Nurse, Tarporley, Cheshire, thanks those who have answered her appeal for "Home for Incurable Girl." All the terms named are too high, 5s. weekly being the only payment which can be made.

A lady will be glad to hear of a home for a child of six and a half. The mother is a poor widow with a younger child to support. Particulars can be obtained from Miss Townsend, 28, Campden Grove, London.

A correspondent will be grateful to anyone who can give a letter for the Provident Surgical Appliance Society to a poor and respectable widow. She needs an elastic stocking to enable her to continue her work as cleaner of offices.

### Some Australian Experiences.

(*Continued from page* clxviii.)

### AT THE GOLD FIELDS.

By the end of November both male and female wards were full, also my own room, which I gave up to very bad cases, and from that time till March I never went to bed in a natural manner; just got snatches of sleep in a chair in one of the wards, the male ward generally, where I had my typhoid case, and a very bad one of gulf fever. At another time I just spread a blanket on the verandah and lay on that. The insects were fearful just before the rains; they came in clouds, and the mosquitoes were particularly vicious. There is also a most abominable rash called "prickly heat," just like tens of thousands of pin points going into you at once, so that all things combine to make life rather trying.

The anxiety of the work was fearful. I tried in all directions to get a goat, just for a little fresh milk for my typhoid, and after many unsuccessful attempts, one morning a little girl appeared with a nannie on a string, with a note from a woman, who informed me I might have it for 15s., as it was dying from starvation, so I took her, and after a few warm bran mashes and some linseed she revived, and to my delight yielded about a breakfast cup of new milk, so I eked it out for my three worst cases. The bad typhoid case was a great favourite with his mates, and a whole lot of the "boys" used to come every evening to make inquiries, so I used to say, "get me some fresh milk or he will die." At length, early one morning, one of them appeared with a bottle of milk, but said I was not to ask where it came from; I took it, and a good many more afterwards, but to this day I have a shrewd suspicion that someone's goats suffered; but my people began to mend, and I did not lose a case from November till April, the worst time of the year.

The numbers varied from seventeen to twenty, by which you will see I had a busy time of it. We had to carry all the water from the tanks up four steps for sponging and washing purposes, and many times I have had to scrub the ward myself, and I always washed the extra sheets, pjamas, and towels. The bath-room and lavatories I also always washed out and attended to. Such was my first summer, to say nothing of the wet season, which was a perfect deluge. When I tell you we registered twenty-eight inches of rain in twenty-four hours you will be able to form some slight idea of the fall we had. The inconvenience during this time is simply indescribable; the water beat in at every turn, wards, rooms, and kitchens were flooded, and as soon as it abated we found ourselves cut off from the coast, as all the road to Normanton was under water.

A subscription was raised, and a lifeboat built to go to the assistance of the poor carriers on the roads, but after it was finished and "launched" on a bullock waggon, it had not got more than five miles from the town when waggon, team, boat and all were bogged; for weeks, not even a horse could go on the roads.

The consequence was, provisions ran short—no butter, jam, oatmeal, or indeed anything, it was a fearful time. Fortunately I could give most of my patients beef-tea, and soup, so that rice and beef and bread were all they had. We were like a shipwrecked crew at sea, very much at sea too. However, we struggled on till May, when again the roads were ust fit to get over. Then more trouble was experienced by many poor men who had been caught by the floods, and had to live in trees, subsisting on rats, snakes, and iguanas. Several arrived at the hospital in a semi-imbecile condition from sheer hardship and suffering, and when they recovered from the exhaustion, broke out in boils, &c.

Better times came along though; my wards thinned out, and then I had an attack of the fever, but thanks to the great kindness of several of the gentlemen I got out a good deal, first driving, as I could not sit on horseback, my back was so bad; but as soon as I could I got any amount of riding, and our good, kind doctor encouraged this by always staying at the hospital to give medicines or admit patients in my absence.

I soon knew the "field" very well, and having nursed men from nearly every "camp," could ride to any part of the place in perfect safety alone. Often I got a horse sent up to me to go for a canter when the owner did not need it. I also often went an early morning ride out by five a.m., and home to breakfast at eight a.m.

Towards the end of the winter a few ladies arrived, so we soon formed a cosy little circle; then life was very pleasant, and we actually had a dancing assembly once a fortnight, and both afternoon and moonlight riding parties, to say nothing of the nice little evening "At homes" at one and another's houses, when the gentlemen played whist and the ladies talked, or had music, or else we all got on the verandahs in the glorious moonlight. The moon knows how to shine as well as the sun up in the tropics. All this made up very considerably for the summer's work and anxiety.

We also begun to get a few vegetables. Certainly, we had to pay 1s. per pound for small potatoes and 2s. 6d. for a vegetable marrow or cauliflower, but were glad to get them at any price. Green peas were 1s. per pound in their shells.

Riding gloves of the commonest quality were 7s. 6d., silk ones 5s. 6d., boots 30s. to order, 21s. ready-made. Nothing in the shape of pins, hairpins, &c., under 6d.; and dress material, zephyrs, prints, &c., 1s. 6d. to 5s. per yard; nothing in silk under 9s., and not of the best quality either.

The wet season, sun, and so much dirty work made fearful havoc in my wardrobe. I wore out ten uniform dresses in the first six months, to say nothing of general wear and tear. A pair of boots made to order, costing 30s., lasted me three months, and having to buy my own stout, at 22s. a dozen bottles, I leave you to guess how far my poor salary went.

During the year we had some very good cases. Purulent ophthalmia raged; I had several cases, and got them through without losing one eye, which invoked more night work, as they were dressed every hour, day and night. There were a few accidents through dynamite, which is used for blasting. One of these patients lost an eye. Fractured skulls did remarkably well considering the climate; the men often fight, and are not particular what they use; one old fellow, a Chinaman, had his head battered in with the sharp edge of a candlestick.

Respecting Chinamen, both in the South and up on this goldfield, I always found them very nice to nurse, and so grateful. They are not at all well treated generally, yet they are both civil and industrious. We should not have had any fresh vegetables or fruit up there had it not been for them. It is so difficult to grow things at all on account of the water being so scarce, yet they carry it great distances for their gardens.

They are very good cooks, too, and in a place where the "servant problem" is difficult to solve, they are a great help.

(*To be continued.*)

### Notes and Queries.

### Queries.

(39) *Training.*—Where can I get the best information on this subject without coming to London?—*Country Bumpkin.*

(40) *District Nurse's Bag.*—Will some one kindly advise me where to get a bag? I want one not too heavy to carry on my rounds, which are often long ones, and I cannot afford to spend much money.—*Nurse Ellen.*

(41) *Age.*—At what age are probationers admitted to King's College Hospital?—*Muriel.*

### Answers.

(39) *Training* (*Country Bumpkin*).—Write to Scientific Press, 140, Strand, London, for "How to Become a Nnrse," by Honnor Morten, price 2s. 6d.

(40) *District Nurse's Bag* (*Nurse Ellen*).—If you will read the notice in last week's HOSPITAL you will find that Allen and Hanbury keep excellent bags of all sizes at moderate prices.

(41) *Age* (*Muriel*).—Write to the Matron and inquire.

